# The Causal Relationship Between Long-Term Exposure to Major PM_2.5_ Constituents and the Rate of Emergency Department Visits: A Difference-in-Differences Study

**DOI:** 10.3390/toxics13110973

**Published:** 2025-11-12

**Authors:** Peizhen Zhao, Chenxi Xie, Shenghao Wang, Shao Lin, Guanghui Dong, Jiashun Li, Sen Yu, Ting Zhang, Xiaozhou Yu, Xian Lin, Sizhe Li, Xiaoru Wu, Jiyuan Zhou, Wangjian Zhang

**Affiliations:** 1 Department of Sexually Transmitted Diseases Control, Dermatology Hospital, Southern Medical University, Guangzhou 510091, China; zhaopeizhen89@i.smu.edu.cn; 2 Department of Biostatistics, School of Public Health (State Key Laboratory of Multi-Organ Injury Prevention and Treatment, and Guangdong Provincial Key Laboratory of Tropical Disease Research), Southern Medical University, Guangzhou 510515, China; a22320064@i.smu.edu.cn; 3 The Eighth Affiliated Hospital, Sun Yat-sen University, Shenzhen 518000, China; wangshh88@mail.sysu.edu.cn; 4 Department of Environmental Health Sciences, School of Public Health, University at Albany, State University of New York, Rensselaer, NY 12222, USA; slin@albany.edu; 5 Department of Occupational and Environmental Health, School of Public Health, Sun Yat-sen University, Guangzhou 510080, China; donggh5@mail.sysu.edu.cn; 6 Department of Medical Statistics, School of Public Health & Center for Health Information Research & Sun Yat-sen Global Health Institute, Sun Yat-sen University, Guangzhou 510080, China; lijsh73@mail2.sysu.edu.cn (J.L.); yusen@mail2.sysu.edu.cn (S.Y.); zhangt528@mail2.sysu.edu.cn (T.Z.); yuxzh@mail2.sysu.edu.cn (X.Y.); linx66@mail2.sysu.edu.cn (X.L.); liszh27@mail2.sysu.edu.cn (S.L.); wuxr35@mail2.sysu.edu.cn (X.W.)

**Keywords:** emergency department visit, PM_2.5_ constituents, difference-in-differences, gWQS, socioeconomic heterogeneity

## Abstract

Fine particulate matter (PM_2.5_) is a well-established health hazard, yet population-level causal evidence on the long-term effects of its chemical constituents and their interactions with environmental and socioeconomic factors remains scarce. This study leveraged quasi-experimental variation in PM_2.5_ exposure across Guangdong province, China, during 2007–2018 to evaluate its causal impact on emergency department (ED) visits. We applied a Difference-in-Differences (DID) causal inference framework to obtain counterfactual estimates of long-term exposure effects and complemented this with generalized Weighted Quantile Sum (gWQS) regression to treat PM_2.5_ as a complex mixture, quantify joint effects, and identify toxic components. The results showed that each interquartile increase in long-term PM_2.5_ exposure was associated with a 10.2% rise in ED visits, with nitrate (weight = 0.299) and sulfate (0.294) contributing the most strongly, while organic matter exerted greater effects in less-developed regions. Temperature variation further modified these effects, with a 1 °C increase in average summer temperature associated with a 3.3% increase and a decrease in winter temperature linked to a 0.54% increase in constituent-related ED visits. Socioeconomic stratification revealed heterogeneous toxicity profiles across regions. These findings provide robust causal evidence on constituent-specific risks of PM_2.5_, highlight the utility of integrating causal and mixture methods for complex exposures, and support targeted emission control and climate-adaptive strategies to protect vulnerable populations.

## 1. Introduction

In 2019, air pollution was estimated to cause approximately 6.7 million premature deaths globally, of which fine particulate matter (PM_2.5_) accounted for 4.14 million [1]. Since 2019, PM_2.5_ concentrations in China have continued to decline due to strengthened clean air policies, yet the total health burden attributable to PM_2.5_ has not shown a consistent reduction because of population aging and the high sensitivity of those with cardiopulmonary diseases to chronic exposure [2,3]. PM_2.5_ exposure has been linked to various adverse health outcomes, including stroke [4], ischemic heart disease [5], asthma [6], adverse pregnancy outcomes [7], and chronic sinusitis [8], among others [9,10,11]. Despite its recognition as a dominant environmental hazard, several key challenges hinder effective public health interventions [12].

First, most studies emphasize short-term PM_2.5_ exposure, focusing on mortality or hospitalization risks [13,14]. However, long-term exposures often bear greater public health importance. Emergency department (ED) visits, as a more sensitive indicator of disease burden than hospitalizations or deaths, capture a broader spectrum of health impacts [15], particularly in low- and middle-income countries where emergency care strain remains high. Moreover, emergency department visits encompass a wide range of acute health events, providing a comprehensive reflection of the impact of air pollution on the health of different population groups [16,17]. Annual PM_2.5_ concentrations reflect year-to-year variations, such as those arising from policy interventions, and prior evidence suggests that even emergency or outpatient visits may be affected by long-term exposure, particularly among chronic or respiratory-sensitive populations [18,19]. Previous studies have also adopted longer temporal exposure windows to investigate the relationship between long-term PM_2.5_ exposure and emergency department visits [20,21]. In our study, we assume that years with higher annual PM_2.5_ concentrations are associated with correspondingly higher cumulative counts of emergency department visits, allowing us to examine the impact of long-term exposure at the population level.

Second, research typically treats PM_2.5_ as a homogeneous mixture, overshadowing compositional heterogeneity. PM_2.5_ comprises chemically distinct aerosols—including black carbon (BC), organic matter (OM), sulfates, ammonium, and nitrates [22]—which may exert divergent pathophysiological effects. For instance, nitrates can provoke inflammatory responses involving IL-6 [23]; black carbon facilitates pulmonary deposition of toxins and oxidative stress [24,25]; ammonium may modulate immune signaling via Th1/Th2/Th17 pathways [26]; and other components act via distinct mechanisms [27,28]. This underscores the need for regionally tailored interventions, yet current global policies remain largely based on total PM_2.5_ mass, potentially misallocating limited resources.

Finally, conventional epidemiological models frequently suffer from residual confounding and cannot establish causality, especially when components are intercorrelated [29,30,31]. Mixture analysis is essential to quantify individual and joint component effects in complex exposures.

To directly address these research gaps, this study aims to: (1) quantify the long-term effects of annual PM_2.5_ exposure on emergency department (ED) visit rates, a sensitive indicator of disease burden; (2) disentangle the heterogeneous health effects of major PM_2.5_ chemical components—black carbon, organic matter, sulfate, nitrate, and ammonium—within a mixture framework; and (3) examine whether socioeconomic disparities modify these associations. To address these research gaps, we employ a Difference-in-Differences (DID) causal inference framework with a generalized Weighted Quantile Sum (gWQS) regression model. The gWQS model effectively mitigates multicollinearity by combining quantile transformation with mixture dimensionality reduction and has become a widely adopted approach in environmental epidemiology for assessing multi-pollutant exposures [31,32]. This combined approach allows us to disentangle the contributions of individual PM_2.5_ chemical components and their combined mixture effects while also strengthening causal interpretation by accounting for time-invariant confounders and exposure correlations. Collectively, this framework provides a more robust and policy-relevant understanding of how long-term and compositional variations in PM_2.5_ influence population-level healthcare demand. We further examine the modifying influences of socioeconomic indicators (per capita GDP, healthcare personnel allocation, urbanization rate) and climatic factors (temperature), advancing epidemiological understanding, refining health risk assessments for susceptible populations, and providing evidence critical for targeted environmental health policy. By identifying the most harmful PM_2.5_ constituents and their context-specific impacts, our findings can inform regionally tailored emission control strategies, support the integration of climate adaptation into air quality management, and guide resource allocation to reduce health inequalities.

## 2. Materials and Methods

### 2.1. Study Settings and the Outcome Data

We obtained city-level emergency department visit data from the Guangdong Department of Health [33]. Guangdong is a large and the most populated province in southern China, and it has a population exceeding 126 million as of 2020, accounting for 8.9% of the country’s total population while covering only 1.8% of its land area [34]. In recent decades, Guangdong has faced severe air pollution, accompanied by a rising rate of ED visits [33,35]. We defined the outcome as the annual number of emergency department visits for each city between 2007 and 2018.

### 2.2. Exposure Data

We obtained daily concentrations of PM_2.5_ and its components at a 10 km × 10 km spatial resolution for 2007–2018 from the Tracking Air Pollution (TAP) dataset [36]. The TAP dataset integrated ground-based PM_2.5_ observations, land use data, population data, and other sources to comprehensively estimate the concentrations of PM_2.5_ and its components [36]. Specifically, TAP defined a high pollution index and employed the SMOTE oversampling technique to balance the sample sizes between high-pollution and normal regions. The SMOTE-oversampled data was then processed using a two-stage random forest model to simulate the concentrations of PM_2.5_ exposure data. The performance of the model was confirmed with a cross-validation R^2^ of 0.83 for overall PM_2.5_ concentrations. The R^2^ values for its components including BC, OM, nitrate, sulfate, and ammonium with R^2^ values of 0.64, 0.72, 0.70, 0.75, and 0.75, respectively [37]. The TAP database has been widely utilized in previous studies [38,39], and detailed information about the database has been published elsewhere [36,37]. We calculated the annual average concentrations of PM_2.5_ and its components for each city by averaging the original values across all grids within the city for each year.

### 2.3. Confounder Data

We obtained daily mean temperature data from 2007 to 2018 from the China Meteorological Forcing Dataset (CMFD), featuring a spatial resolution of 0.1° × 0.1° [40]. To determine the daily mean temperatures for each city, we averaged the temperature values from all grid points within a city. We then calculated the seasonal mean temperatures and their standard deviations (SDs) for summer (from June to August) and winter (from December to February).

We obtained socioeconomic data from the Guangdong Province Statistical Yearbook, which includes information on per capita GDP, urbanization level (proportion of people living in urban areas), and the number of healthcare professionals per thousand people.

### 2.4. Statistical Method

We employed a Difference-in-Differences (DID) method based on an extension of the Rubin Causal Model to handle confounders across multiple spatial and temporal contexts [41,42]. Confounders are classified into three categories: those associated solely with time, those associated solely with space, and those that vary with both time and space. This approach enhances the traditional DID method by addressing time- and space-specific confounders through comparisons of differences between adjacent years across different regions. Under this approach, the only confounders that need to be controlled are those that vary simultaneously with time and space, such as temperature and GDP per capita. The DID model was specified as:

**Model 1**

$$\ln\left(E\left(Y_{s,t}\right)\right)=\beta_{0}+\beta_{1}I_{s}+\beta_{2}I_{t}+\beta_{3}{Component}_{s,t,i}+\beta_{4}{Tsummer}_{s,t}+\beta_{5}{Twinter}_{s,t}\;\;\;\;\;\;\;\;\;\;\;\;\;\;\;\;\;\;\;\;\;\;\;\;\;\;\;\;\;\;\;\;\;\;\;+\beta_{6}{SD\_Tsummer}_{s,t}+\beta_{7}{SD\_Twinter}_{s,t}+\beta_{8}{GDP}_{s,t}+\ln\left({POP}_{s}\right)$$

In this model, $Y_{s,t}$ represents the count of visits to the ED in region *s* during year *t*. $I_{s}$ and $I_{t}$ are indicator variables for regions and years, respectively. ${Component}_{s,t,i}$ refers to the components of PM_2.5_, such as sulfate or organic matter. ${Tsummer}_{s,t}$, ${SD\_Tsummer}_{s,t}$, ${Twinter}_{s,t}$, and ${SD\_Twinter}_{s,t}$ represent the average temperature and standard deviation during summer and winter, in that order. ${GDP}_{s,t}$ indicates the GDP per capita of region *s* in year *t*. To validate the parallel trend assumption of the DID model [41,42], we observed a minimal correlation between changes in confounding factors and exposure rates following previous studies [42]. As for the link function of the DID model, we chose Poisson regression. The impact of exposure on outcomes was measured by the percentage increase in risk for each IQR increase in exposure level.

We applied the gWQS_add_ model to explore the combined effects of PM_2.5_ and its components on the outcome. We combined the gWQS regression with the DID model, using quantile weighting of the independent variables to construct a WQS index, which reduces dimensionality and avoids issues of multicollinearity that traditional methods face when handling exposure [43]. In calculating the WQS index, gWQS_add_ computes the weight of each variable, reflecting their relative contributions to the WQS index. The gWQS_add_ model in our study was specified as follows:

**Model 2**

$$\ln\left(E\left(Y_{s,t}\right)\right)=\beta_{0}+\beta_{1}I_{s}+\beta_{2}I_{t}+\beta_{3}\sum_{i=1}^{c}w_{i}q_{i}+\beta_{4}{Tsummer}_{s,t}+\beta_{5}{Twinter}_{s,t}\;\;\;\;\;\;\;\;\;\;\;\;\;\;\;\;\;\;\;\;\;\;\;\;\;\;\;\;\;\;\;\;\;\;\;\;\;\;\;\;\;\;\;\;\;\;\;\;\;+\beta_{6}{SD\_Tsummer}_{s,t}+\beta_{7}{SD\_Twinter}_{s,t}+\beta_{8}{GDP}_{s,t}+\ln\left({POP}_{s}\right)|b$$

In this model, $q_{i}$ represents the quantile of component *i*, and $w_{i}$ denotes the weight of the component *i*. Let *b* be the number of bootstrap resamples. The ${WQS}_{add}$ index is defined as $\sum_{i=1}^{c}w_{i}q_{i}$. Based on previous research, all five components of PM_2.5_ were designated as risk factors [44]; thus, a positive weight constraint was applied when estimating the weight parameters via bootstrap resampling. In this study, components with $w_{i}$ values exceeding 0.2 were classified as key components [43].

We also explored the interaction between summer and winter temperature changes and PM_2.5_ by incorporating interaction terms between summer temperature and the WQS index, as well as between winter temperature and the WQS index, in the gWQS_add_ model [42].

We used the gWQS_int_ model to investigate whether the effects of PM_2.5_ vary across different socioeconomic strata [45]. We classified annual GDP per capita, healthcare personnel allocation, and urbanization rate into high and low categories according to their comparison with the mean values. The gWQS_int_ model is as follows:

**Model 3**

$$\ln\left(E\left(Y_{s,t}\right)\right)=\beta_{0}+\beta_{1}I_{s}+\beta_{2}I_{t}+\beta_{3}(\sum_{i=1}^{c}w_{i}q_{i}+\sum_{i=c+1}^{c+l}w_{i}s_{i})+\beta_{4}{Tsummer}_{s,t}+\beta_{5}{Twinter}_{s,t}\;\;\;\;\;\;\;\;\;\;\;\;\;\;\;\;\;\;\;\;\;\;\;\;\;\;\;+\beta_{6}{SD\_Tsummer}_{s,t}+\beta_{7}{SD\_Twinter}_{s,t}+\beta_{8}{GDP}_{s,t}+\ln\left({POP}_{s}\right)|b$$

In this model, $s_{i}$ represents the interaction terms, where each economic indicator variable was separately added to the model. The ${WQS}_{int}$ index is defined as $\sum_{i=1}^{c}w_{i}q_{i}+\sum_{i=c+1}^{c+l}w_{i}s_{i}$. Typically, the coefficients for the interaction terms between socioeconomic indicators and PM_2.5_ may not always be positive. Therefore, we use either higher or lower categories as references to fit the model, performing two directional fittings. We use specific-stratum weight (SSW) to represent the relative contribution of each component and reweight it as ${SSW}_{ij}=w_{ij}/\sum_{i=1}^{c}w_{ij}$ [31]. ${SSW}_{ij}$ reflects the SSW of component *i* in group *j*, under a given socioeconomic indicator.

We performed a sensitivity analysis of the results. First, we replaced the main model with the annual moving average concentrations of pollutants for 0–1 and 0–2 years to calculate the lag effects of each component and combined exposure. Second, we used the quintile and decile methods instead of the quartile method used in the main analysis to assess the influence of different categorization strategies on the results. Third, we incorporated a spatial lag term, constructed from the Queen contiguity matrix, into Model 1 to re-estimate the effects of the five PM_2.5_ components and the WQS_add_ index, in order to evaluate the potential impact of spatial autocorrelation.

## 3. Results

### 3.1. Descriptive Analysis

From 2007 to 2018, a total of 61.536 million ED visits were reported in the study area. During this period, the average concentrations of exposure (PM_2.5_, nitrates, sulfates, ammonium salts, OM, and BC) were 7.365 (± 2.472) μg/m^3^, 34.685 (± 11.256) μg/m^3^, 5.438 (± 1.341) μg/m^3^, 4.500 (± 1.179) μg/m^3^, 9.471 (± 2.943) μg/m^3^, and 2.209 (± 0.786) μg/m^3^, respectively (Table 1). We observed high correlations among concentrations of the components, with correlation coefficients ranging from 0.86 to 0.99 (*p* < 0.05) (Figure S1).

### 3.2. Causal Associations of PM_2.5_ and Its Components with Emergency Department Visits

Our study identified a significant correlation between PM_2.5_ and its components and ED visit rates (*p* < 0.001). As displayed in Table 2, each IQR increase in PM_2.5_ concentration may result in a 10.198% (95% CI: 10.171%, 10.225%) increase in the rate of ED visits. Similarly, we observed increases in the rate of ED visits associated with each IQR increase in the concentrations of PM_2.5_ components: 10.729% (95% CI: 10.698%, 10.761%) for nitrate, 11.415% (95% CI: 11.388%, 11.443%) for sulfate, 10.921% (95% CI: 10.887%, 10.955%) for ammonium, 10.688% (95% CI: 10.661%, 10.716%) for OM, and 11.756% (95% CI: 11.726%, 11.787%) for BC.

**Figure 1:**
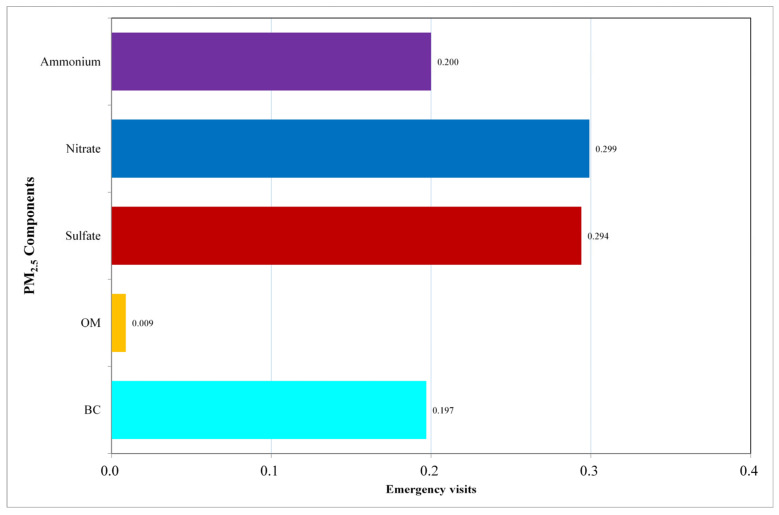
Contribution of Each Component to the WQS_add_ Index. This figure presents the contribution weights of the five PM_2.5_ components—Black Carbon, Organic Matter, Sulfate, Nitrate, and Ammonium—to the Weighted Quantile Sum (WQS) Index.

The outcomes of the gWQS_add_ model are presented in Figure 1. We found that every unit increment in the WQS_add_ index, which reflects the combined effect of the components mixture, was linked to a 7.56% (95% CI: 7.53%, 7.59%) rise in the rate of ED visits. Among these components, nitrates (weight: 0.299) and sulfates (weight: 0.294) are the most influential, followed by ammonium (weight: 0.2), BC (weight: 0.197), and organic matter (weight: 0.009).

### 3.3. The Interaction of Temperature and PM_2.5_ on Emergency Visits

An interaction effect was found between the average temperatures in winter and summer and the combined exposure to PM_2.5_ (Table 3). At the average winter and summer temperatures across all regions and years, each unit increase in the ${gWQS}_{add}$ index corresponds to an IR% of 6.259% (95% CI: 5.918%, 6.601%) in the rate of ED visits. When the summer average temperature rises by 1 °C above the mean, every unit increment in the WQS_add_ index corresponds to a 3.298% higher IR% of ED visits, resulting in an updated IR% of 9.557% (P_interaction_ < 0.001). When the average winter temperature decreases by 1 °C below the average, every unit increment in the WQS_add_ index may result in a 0.54% greater IR% of ED visits, with an updated IR% of 6.799% and P_interaction_ of 0.028.

### 3.4. The Modifying Effects of Socioeconomic Factors

**Figure 2:**
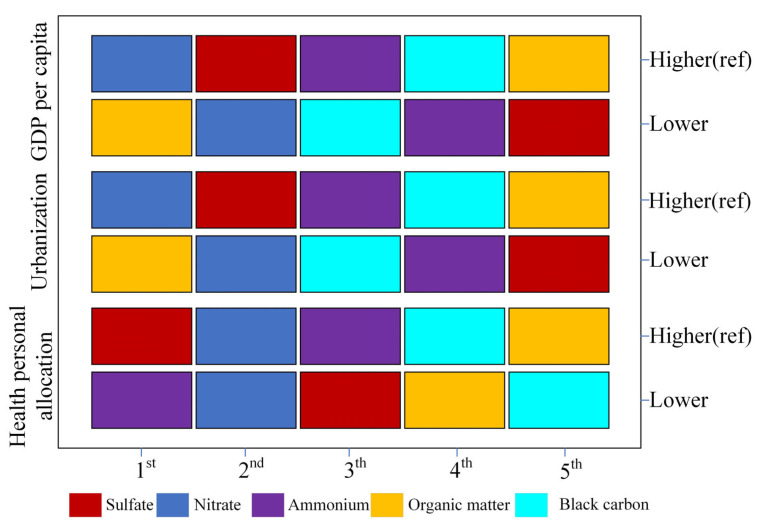
Ranking of Specific Stratified Weights (SSWs) for PM_2.5_ Components. This figure illustrates the ranking of specific stratified weights (SSWs) for PM_2.5_ components under different socioeconomic levels (including health personal allocation, urbanization, and GDP per capita).

Figure 2 and Figure S2 illustrate the moderating effect of socioeconomic factors on PM_2.5_. The results across different socioeconomic strata are generally consistent with those observed for the entire population, with nitrates and sulfates playing critical roles in nearly all strata. Specifically, in regions with higher levels of socioeconomic status, nitrates (SSW range: 0.199 to 0.294) and sulfates (SSW range: 0.189 to 0.443) displayed stronger effects. However, in regions with lower GDP (SSW: 0.220) and urbanization rates (SSW: 0.202), organic matter tended to be the most influential among PM_2.5_ components (Table S2).

In sensitivity analysis, despite some variations in effect sizes (IR%) when using 1–2 year lagged data or applying quintiles and deciles instead of quartiles, or incorporating a spatial lag term constructed from the Queen contiguity matrix into Model 1 to account for potential spatial autocorrelation, our findings remain unchanged (Table S3).

## 4. Discussion

Our study revealed a significant positive association between PM_2.5_, its components, and emergency department visits, with a particular emphasis on the substantial contribution of sulfate. Additionally, our subgroup analysis revealed significant effects of nitrate and sulfate across nearly all strata, followed by ammonium. In regions with lower socioeconomic status, the correlations were more pronounced for organic matters than other components. This study also explored the interaction between temperature and the PM_2.5_ mixture, suggesting that both increases in average summer temperatures and decreases in average winter temperatures may exacerbate the impact of the PM_2.5_ mixture.

Because PM_2.5_ components were highly correlated (r = 0.86–0.99), the single-component DID models exhibited large variance inflation factors (VIF > 10), indicating potential multicollinearity and unstable estimates (Table S4). To address this issue, we employed DID-based gWQS regression in a complementary manner to single-component DID models. The gWQS approach transforms exposures into quartiles and applies bootstrap weighting to mitigate collinearity while simultaneously quantifying the overall mixture effect and the relative contribution of each component, thereby complementing the straightforward interpretation provided by single-component analyses.

We observed that nitrate and sulfate are the components most closely associated with increased emergency department visits. Previous studies have also reached similar conclusions, which suggested significant associations of nitrate and sulfate with a variety of diseases, such as cardiovascular diseases [46], chronic obstructive pulmonary disease [47], asthma [48], and others [49]. For instance, a study in California found that nitrate and sulfate in PM_2.5_ contributed to increased IR% of respiratory diseases such as asthma (IR%: 3.3%, 95% CI: 1.1%,5.5%) and bronchitis (IR%:3.0%, 95% CI: 0.4%,5.7%) [50]. Experimental studies suggested that nitrate and sulfate in PM_2.5_ particles could penetrate the pulmonary surfactant barrier and interfere with gene expression in lung cells [28], leading to neutrophil infiltration in the lungs which further impairs respiratory function [51]. Nitrate and sulfate could also penetrate the placenta barrier and result in various adverse pregnancy outcomes [52] which is also a significant part of emergency department visits. Compared with these findings from the gWQS model, the results from the DID model were different, which showed a significant health impact of black carbon (BC) rather than nitrate. This disparity was commonly reported in previous studies [53,54]. The use of the gWQS model, as a mixture analysis method, enables the investigation of the joint effects of PM_2.5_ component mixtures. This model addresses the limitations of single-component DID models, which can only consider one component at a time and cannot account for the high correlations among components. However, evidence regarding the mechanisms through which different components affect health remains scarce, necessitating further research to elucidate their underlying pathways.

${NH}_{4}^{+}$ was also identified as having significant health effects in the current research. Research has demonstrated that ${NH}_{4}^{+}$ is linked to various diseases, such as idiopathic pulmonary fibrosis (ILD) (HR per decile increase: 1.38, 95% CI: 1.36, 1.40), asthma (OR per standard deviation increase: 1.28, 95% CI: 1.17, 1.41), and atherosclerotic cardiovascular diseases (OR per standard deviation increase:1.21, 95% CI: 1.10,1.32) [48,53,54]. The potential mechanisms underlying the health impact of ammonium salts include inducing systemic inflammation, disrupting the balance between regulatory T cells and TH1 cells, activating the NF-κB pathway to trigger respiratory inflammation, and may activate mast cells and basophils, potentially playing a key role in asthma [26,55]. In the Pearl River Delta region, non-agricultural sources account for 63.74% of ammonium salts. The main contributors include biomass burning (12.71% ± 3.63%), coal combustion (14.70% ± 5.38%), vehicle emissions (14.24% ± 5.55%), and waste (22.09% ± 12.48%) [56]. Therefore, the existing environmental policies should be expanded to cover these sources to effectively reduce the disease burden.

Our study found that organic matter had the most significant health effects among the five components in regions with lower socioeconomic status. This result aligns with findings from previous studies. For instance, Cai et al. reported that organic matter had a particularly strong impact on stroke outcomes in regions with relatively lower economic development, with an interquartile range increase of 3.47 μg/m^3^ in organic matter associated with an odds ratio (OR) of 1.086 (95% CI: 1.069–1.104) for stroke fatality among 3,069,093 hospitalized patients [57]. This finding may be attributed to differences in multiple aspects such as pollution sources and medical services across regions [58,59,60]. In economically disadvantaged regions, a substantial proportion of households continue to rely on traditional solid fuels such as wood, coal, and crop residues like straw for cooking and heating [61,62]. The incomplete combustion of these organic materials not only produces elevated levels of particulate matter but also generates a complex mixture of organic pollutants, including polycyclic aromatic hydrocarbons (PAHs), volatile organic compounds (VOCs), and other toxic by-products, which can lead to significant health hazards [63,64,65,66]. Some studies suggest that the use of biomass fuels in low-income regions generates significant amounts of PM_2.5_ [62,67]. A study in Chicago found that low-income households had significantly higher indoor PM_2.5_ levels compared with high-income households (52.5 μg/m^3^ vs. 18.2 μg/m^3^), indicating that economically disadvantaged households are exposed to elevated indoor air pollution. This suggests that in lower-SES regions, indoor sources may amplify the health effects of PM_2.5_ organic matter and increase the risk of emergency department visits [68]. Due to the lack of effective management and residents’ tendency to spend more time indoors, exposure levels are higher, thereby increasing the risk of disease burden [69,70,71,72]. Insufficient sanitation facilities, lack of health awareness and medical services, and poor management of chronic diseases in these regions may also contribute to a higher rate of ED visits [73,74,75]. Socioeconomic position (SEP) not only influences overall health but may also play a role in disease and mortality related to air pollution. Low-income or disadvantaged populations are often exposed to higher levels of air pollution and may be more susceptible due to interactions with other harmful exposures or chronic health conditions [76,77]. Therefore, it is crucial to further investigate the impacts of PM_2.5_ on health across various socioeconomic levels. Though the mechanisms remain incompletely understood, controlling the combustion of organic matter and adopting cleaner energy sources, such as natural gas and clean coal, could improve the health of residents in economically challenged areas.

Our study shows that temperature affects the effects of PM_2.5_ on health, with an increase in summer average temperatures and a decrease in winter average temperatures both leading to higher emergency department visit rates. Research by Yitshak-Sade et al. found that fluctuations in temperature and the increase in extreme weather events could interact with air pollutants, posing risks to human health [78]. Studies on PM_2.5_ and asthma have shown that within a temperature range of 1.1–44.4 °F, each 1 μg/m^3^ rise in PM_2.5_ concentration was linked to a 7.9% increase in the incidence of asthma; in the 44.5–58.6 °F range, it was 6.9%; in the 58.7–70.1 °F range, 2.9%; and in the 70.2–80.5 °F range, 7.3% [79]. A study in Guangzhou found that under low- and high-temperature conditions, each 10 μg/m^3^ increase in PM_2.5_ concentration led to a 0.73% and 0.46% rise in non-accidental mortality rates, respectively [80]. Higher temperatures and humidity levels in the summer can make the population more sensitive to the health burden caused by PM_2.5_ [81,82], probably by exacerbating the oxidative stress and inflammatory responses induced by the particles [83]. Higher temperatures may also enhance atmospheric photochemical reactions, promoting the formation of secondary aerosols such as nitrates and sulfates, thereby increasing the health burden [84]. The decrease in winter temperature enhances the body’s response to air pollutants [85]. The low temperature further promotes the reaction between ${SO}_{2}$ and OH radicals, leading to the formation of ${SO}_{4}^{2-}$ [86]. Additionally, the drop in winter temperatures can weaken immune function [87]. A large body of research has shown that PM_2.5_ exposure not only leads to increased arterial blood pressure but is also associated with elevated levels of C-reactive protein, which in turn exacerbates the disease burden [88,89]. Our findings align with numerous previous studies; however, the interactions between PM_2.5_ and temperature exhibit variability across different regions. The mechanisms underlying this interaction need further investigation. A study found that both heat waves and cold spells can modify the health effects of PM_2.5_ constituents on stroke mortality. Heat waves exhibited pronounced synergistic interactions with secondary inorganic aerosols (NO_3_^−^, SO_4_^2−^, NH_4_^+^), substantially increasing the risk of stroke death, whereas the interactions during cold spells were weaker, with risk estimates ranging from 1.19 to 1.55 for heat waves and 1.03–1.11 for cold spells [90].

Our findings indicate that the PM_2.5_ components most strongly associated with adverse health impacts—particularly nitrate and sulfate—are mainly linked to traffic and industrial emissions, while organic matter poses greater risks in less economically developed regions. These results underscore the need for source-oriented emission control strategies to reduce long-term exposure risks. Consistently, recent policy simulations in Guang-dong demonstrate that structural interventions—including industrial upgrading, strict-er vehicle emission standards, and cleaner energy transitions—are more effective in reducing harmful PM_2.5_ components than end-of-pipe controls alone [91]. To further mitigate exposure and its associated health burden, current environmental policies should be expanded to incorporate green infrastructure, such as urban street trees and vegetation barriers, which have been shown to reduce traffic-related pollution [92,93]. Moreover, policy evaluations in Guangdong highlight that socioeconomic and institutional conditions critically shape PM_2.5_ emission patterns. Increasing marketisation and advancing industrial transformation can curb pollution from traditional manufacturing, while globalization-driven clean technology adoption and stringent environmental regulation effectively reduce emissions from pollution-intensive industries [94]. Conversely, decentralization without adequate regulatory oversight may exacerbate industrial pollutant discharge, reinforcing the importance of coordinated cross-city governance to address spatial spillover effects within the Pearl River Delta [95]. Together, these insights support multi-dimensional strategies that integrate economic restructuring, regulatory enhancement, and green urban development to reduce harmful PM_2.5_ components and their health impacts. Our study found that organic matter in PM_2.5_ has the greatest impact on ED visits in less economically developed regions. This aligns with evidence from rural China, where household use of solid fuels such as wood and crop residues generates high levels of indoor organic particles, particularly in kitchens and bedrooms, posing greater health risks for residents who spend more time indoors, such as elderly women [96]. Therefore, in addition to controlling traffic and industrial emissions, promoting cleaner household energy and improving indoor air quality are crucial strategies to reduce the health burden of PM_2.5_ in economically disadvantaged areas.

Our study boasts several strengths, including the combination of the causal inference and the mixture analysis approaches, and being among the few studies elucidating the causal links between PM_2.5_ components mixture and the rate of emergency department visits. However, we still need to interpret our findings cautiously. Firstly, the absence of detailed individual-level data restricts the capacity to control for individual-level confounders. Secondly, due to the absence of high-resolution PM_2.5_ and component data, we relied on simulated TAP data at 10 × 10 km resolution, which may overlook within-city exposure differences. Due to the limited spatial resolution of TAP exposure data, some exposure misclassification is inevitable; however, this is expected to be non-differential across cities, likely biasing the results toward the null rather than generating spurious associations [97]. While these data have been rigorously validated against ground-based measurements, caution is still needed when interpreting the results. While our findings contribute important evidence from Guangdong, further studies conducted in regions with more diverse environmental and sociodemographic characteristics are needed to evaluate the generalizability and robustness of PM_2.5_ component–health associations. Finally, we used the DID model to explore causal effects, but the validity of this approach relies on the parallel trend assumption which could not be directly tested in this multi-year and multi-area design. Following the framework of previous studies, we assessed this assumption by examining low correlation of PM_2.5_ components with potential confounders (Table S1). Despite these limitations, we identified a significant correlation of PM_2.5_ and its components with ED visits.

## 5. Conclusions

This study demonstrated that long-term PM_2.5_ exposure, particularly its nitrate and sulfate components, was strongly associated with increased emergency department visits. We also found that climatic and socioeconomic factors modified these associations, with higher risks under extreme temperatures and greater contributions of organic matter in less-developed regions. These findings provide causal evidence of constituent-specific health risks, highlight the vulnerability of disadvantaged populations, and underscore the need for targeted emission reductions in nitrate and sulfate, alongside clean energy promotion in rural and underdeveloped areas. Integrating air quality management with climate adaptation strategies will be critical to reducing the health burden of PM_2.5_.

## Figures and Tables

**Table 1:** Descriptive Analysis of Emergency Department Visits, PM_2.5_ and Its Components, Temperature, and Socioeconomic Data in Guangdong from 2007 to 2018.

					Percentiles			
Variables	Mean	SD	Min	25th	Median	75th	Max	IQR
Outcome								
Emergency visits (100,000 person times)	24.419	27.439	1.106	6.238	12.957	30.682	114.183	24.445
Environmental data								
Annual PM_2.5_ (μg/m^3^)	34.685	11.256	19.220	27.495	32.110	38.312	89.844	10.817
Annual sulfate (μg/m^3^)	7.365	2.472	4.145	5.738	6.740	8.214	18.881	2.476
Annual nitrate (μg/m^3^)	5.438	1.341	3.106	4.478	5.257	6.121	11.076	1.643
Annual ammonium (μg/m^3^)	4.500	1.179	2.549	3.659	4.272	5.129	9.576	1.470
Annual organic matter (μg/m^3^)	9.471	2.943	5.476	7.567	8.784	10.535	24.259	2.967
Annual black carbon (μg/m^3^)	2.209	0.786	1.195	1.685	2.054	2.500	5.760	0.815
Mean Summer Temperature (°C)	27.842	0.880	25.797	27.261	27.908	28.486	29.670	1.225
SD of Summer T (°C)	1.574	0.278	1.028	1.360	1.531	1.698	2.658	0.338
Mean Winter Temperature (°C)	14.269	2.207	7.362	13.145	14.628	15.825	18.261	2.680
SD of Winter T (°C)	3.788	0.687	2.324	3.237	3.818	4.238	5.437	1.002
Socio-Economic data								
GDP (CNY)	4.341	3.322	0.354	2.180	3.380	5.444	15.532	3.265
Health personnel allocation (per 1000 people)	26.114	25.311	5.781	12.254	17.804	27.366	156.497	15.113
Urbanization Rate (%)	61.506	20.201	34.400	45.000	54.470	84.495	100.000	39.495
Population (thousands)	49.940	28.143	14.544	29.455	41.438	60.906	149.044	31.451

**Table 2:** The results of the single-component DID (Difference-in-Differences) model.

Exposure	IR%	95%CI	*p*
Annual PM_2.5_	10.198	(10.171, 10.225)	<0.001
Annual black carbon	11.756	(11.726, 11.787)	<0.001
Annual organic matter	10.688	(10.661, 10.716)	<0.001
Annual sulfate	11.415	(11.388, 11.443)	<0.001
Annual nitrate	10.729	(10.698, 10.761)	<0.001
Annual ammonium	10.921	(10.887, 10.955)	<0.001

**Table 3:** The mixture effect of PM_2.5_ components under specific summer and winter temperature conditions.

Mean Summer Temperature (°C)	Mean Winter Temperature (°C)	IR%	95% CI	*p* for Interaction
(Average)	(Average)	6.259	(5.918, 6.601)	ref
(Average − 1)	(Average)	3.060	(2.730, 3.392)	<0.001
(Average + 1)	(Average)	9.557	(9.206, 9.910)	<0.001
(Average)	(Average − 1)	6.799	(6.457, 7.143)	0.028
(Average)	(Average + 1)	5.722	(5.383, 6.062)	0.028

## Data Availability

The original contributions presented in this study are included in the article/Supplementary Materials. Further enquiries can be directed to the corresponding author.

## Supplementary Materials

The following supporting information can be downloaded at: https://www.mdpi.com/article/10.3390/toxics13110973/s1, Table S1: Correlation Analysis Between the Relative Rate Differences of PM2.5 Components Concentrations and Those of the Confounders. Table S2: Specific Stratified Weights of PM2.5 Components Across Different Socioeconomic Status Levels. Table S3: Results of Sensitivity Analysis. Table S4: Variance inflation factor (VIF) for multi-exposure DID model. Figure S1: Mean concentration of PM2.5 and mean proportion of 5 PM2.5 constituents in Guangdong, China, 2007–2018. Figure S2: Pearson Correlation Coefficient Matrix Among the Five PM2.5 Components (including organic matter (OM), black carbon (BC), sulfate (SO42−), nitrate (NO3−), and ammonium (NH4+)). Figure S3: Ranking of Specific Stratified Weights (SSWs) for PM2.5 Components (direction 2).
