# Supplementary material for: The Causal Relationship Between Long-Term Exposure to Major PM2.5 Constituents and the Rate of Emergency Department Visits: A Difference-in-Differences Study
Source: Toxics. 2025 Nov 12;13(11):973. doi: 10.3390/toxics13110973 (PMC12656412; doi:10.3390/toxics13110973)
Supplement: Supplementary file 1 [file toxics-13-00973-s001.zip › toxics-3947506-supplementary.pdf]

Supplementary Materials

The Causal Relationship Between Long-Term Exposure to Major $PM_{2.5}$ Constituents and the Rate of Emergency Department Visits: A Difference-in-Differences Study

Peizhen Zhao [1,†], Chenxi Xie [2,†], Shenghao Wang [3], Shao Lin [4], Guanghui Dong [5], Jiashun Li [6], Sen Yu [6], Ting Zhang [6], Xiaozhou Yu [6], Xian Lin [6], Sizhe Li [6], Xiaoru Wu [6], Jiyuan Zhou [2,*] and Wangjian Zhang [6,*]

Author affiliations

[1] Department of Sexually Transmitted Diseases Control, Dermatology Hospital, Southern Medical University, Guangzhou 510091, China

[2] Department of Biostatistics, School of Public Health (State Key Laboratory of Multi-Organ Injury Prevention and Treatment, and Guangdong Provincial Key Laboratory of Tropical Disease Research), Southern Medical University, Guangzhou 510515, China

[3] The Eighth Affiliated Hospital, Sun Yat-sen University, Shenzhen 518000, China

[4] Department of Environmental Health Sciences, School of Public Health, University at Albany, State University of New York, Rensselaer, NY 12222, USA

[5] Department of Occupational and Environmental Health, School of Public Health, Sun Yat-sen University, Guangzhou 510080, China

[6] Department of Medical Statistics, School of Public Health & Center for Health Information Research & Sun Yat-sen Global Health Institute, Sun Yat-sen University, Guangzhou 510080, China

[†] These authors contribute equally to this work

***Corresponding author:**

Jiyuan Zhou, E-mail: zhoujy@smu.edu.cn

Wangjian Zhang, Email: zhangwj227@mail.sysu.edu.cn

1. Parallel Trend Assumption Test

A low correlation was observed between the relative rate difference in each $PM_{2.5}$ component concentration and that of the confounders (Table S1), indicating that the parallel trend assumption was satisfied.

Table S1. Correlation Analysis Between the Relative Rate Differences of $PM_{2.5}$ Components Concentrations and Those of the Confounders.

**Exposures**	**Potential Confounders**		
	**Winter T (°C)**	**Summer T (°C)**	**GDP per Capita (Million)**
Sulfate	0.239 *	0.018	−0.239 *
Nitrate	0.246 *	−0.039	−0.195 *
Ammonium	0.273 *	−0.155	−0.191 *
Organic matter	0.213 *	0.090	−0.202 *
Black carbon	0.205 *	0.107	−0.197 *

* $p < 0.05$.

2. Effect Modification via Socioeconomic Factors

Table S2. Specific Stratified Weights of $PM_{2.5}$ Components Across Different Socioeconomic Status Levels.

gWQSint Model	SSW	
	**Direction 1 (Lowerref)**	**Direction 2 (Upperref)**
Health personnel allocation		
SO_4^{2-} *Lower	0.297	0.197
NO_3^- *Lower	0.286	0.219
NH_4^+ *Lower	0.202	0.229
OM *Lower	0.006	0.184
BC *Lower	0.208	0.172
SO_4^{2-} *Higher	0.221	0.321
NO_3^- *Higher	0.199	0.265
NH_4^+ *Higher	0.199	0.251
OM *Higher	0.185	0.001
BC *Higher	0.195	0.163
GDP per capita		
SO_4^{2-} *Lower	0.284	0.189
NO_3^- *Lower	0.335	0.204
NH_4^+ *Lower	0.206	0.191
OM *Lower	0.011	0.220
BC *Lower	0.164	0.197
SO_4^{2-} *Higher	0.246	0.274
NO_3^- *Higher	0.165	0.286
NH_4^+ *Higher	0.188	0.232
OM *Higher	0.171	0.009
BC *Higher	0.229	0.198
Urbanization Rate		
SO_4^{2-} *Lower	0.214	0.198
NO_3^- *Lower	0.405	0.199
NH_4^+ *Lower	0.170	0.198
OM *Lower	0.015	0.202
BC *Lower	0.196	0.202
SO_4^{2-} *Higher	0.443	0.291
NO_3^- *Higher	0.257	0.294
NH_4^+ *Higher	0.123	0.215
OM *Higher	0.072	0.014
BC *Higher	0.106	0.186

* indicates the effect of the corresponding pollutant at the respective Different Socioeconomic Status Levels.

3. Results of Sensitivity Analysis

Table S3. Results of Sensitivity Analysis.

**Model**	**IR%**	**95% CI**	***p***
Annual BC			
Lag 0–1	12.195	(12.164, 12.226)	<0.001
Lag 0–2	12.486	(12.456, 12.516)	<0.001
Spatial lag	3.880	(3.845, 3.916)	<0.001
Annual OM			
Lag 0–1	11.028	(11.001, 11.055)	<0.001
Lag 0–2	12.436	(12.407, 12.465)	<0.001
Spatial lag	3.880	(3.845, 3.916)	<0.001
Annual sulfate			
Lag 0–1	11.739	(11.711, 11.767)	<0.001
Lag 0–2	12.738	(12.709, 12.767)	<0.001
Spatial lag	3.880	(3.845, 3.916)	<0.001
Annual nitrate			
Lag 0–1	12.543	(12.510, 12.577)	<0.001
Lag 0–2	13.547	(13.513, 13.582)	<0.001
Spatial lag	3.967	(3.933, 4.000)	<0.001
Annual ammonium			
Lag 0–1	12.332	(12.297, 12.367)	<0.001
Lag 0–2	12.700	(12.665, 12.734)	<0.001
Spatial lag	4.466	(4.431, 4.501)	<0.001
WQS Index			
Lag 0–1	8.588	(8.560, 8.616)	<0.001
Lag 0–2	6.642	(6.616, 6.669)	<0.001
Quintile	10.629	(10.606, 10.652)	<0.001
Decile	5.382	(5.369, 5.395)	<0.001
Spatial lag	4.466	(4.431, 4.501)	<0.001

4. Variance Inflation Factor (VIF) for Multi-Exposure DID Model

Table S4. Variance inflation factor (VIF) for multi-exposure DID model.

**Variables**	**VIF**
Annual BC	1463.2
Annual OM	1150.3
Annual SO_4^{2-}	1011.8
Annual NO^{3-}	1124.7
Annual NH^{4+}	1403.5

5. The Mean concentration of $PM_{2.5}$ and mean proportion of 5 $PM_{2.5}$ constituents—Black Carbon, Organic Matter, Sulfate, Nitrate, and Ammonium.

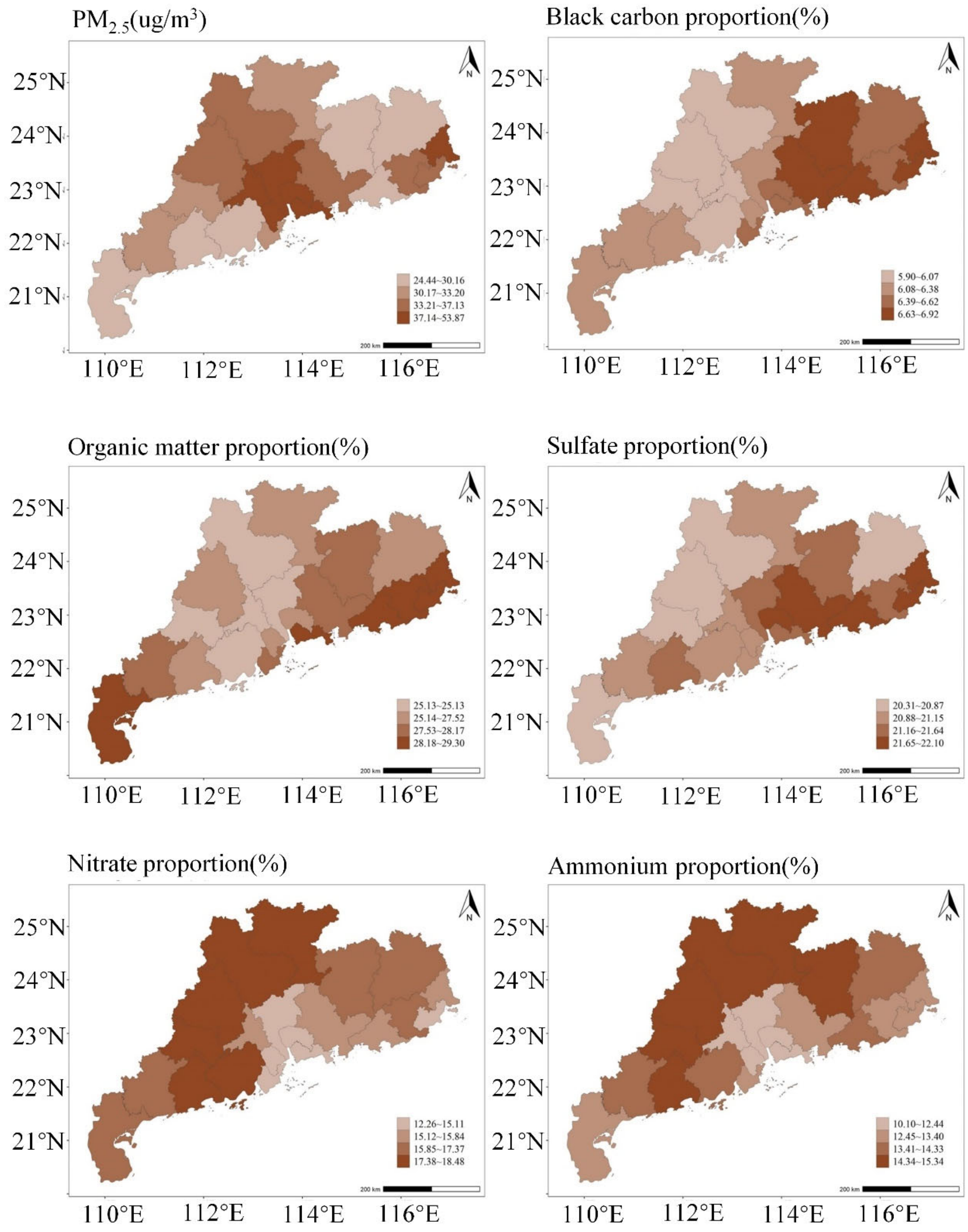

Figure S1 Mean concentration of $PM_{2.5}$ and mean proportion of 5 $PM_{2.5}$ constituents in Guangdong, China, 2007–2018.

6. Pairwise Correlation Analysis of the Five Components

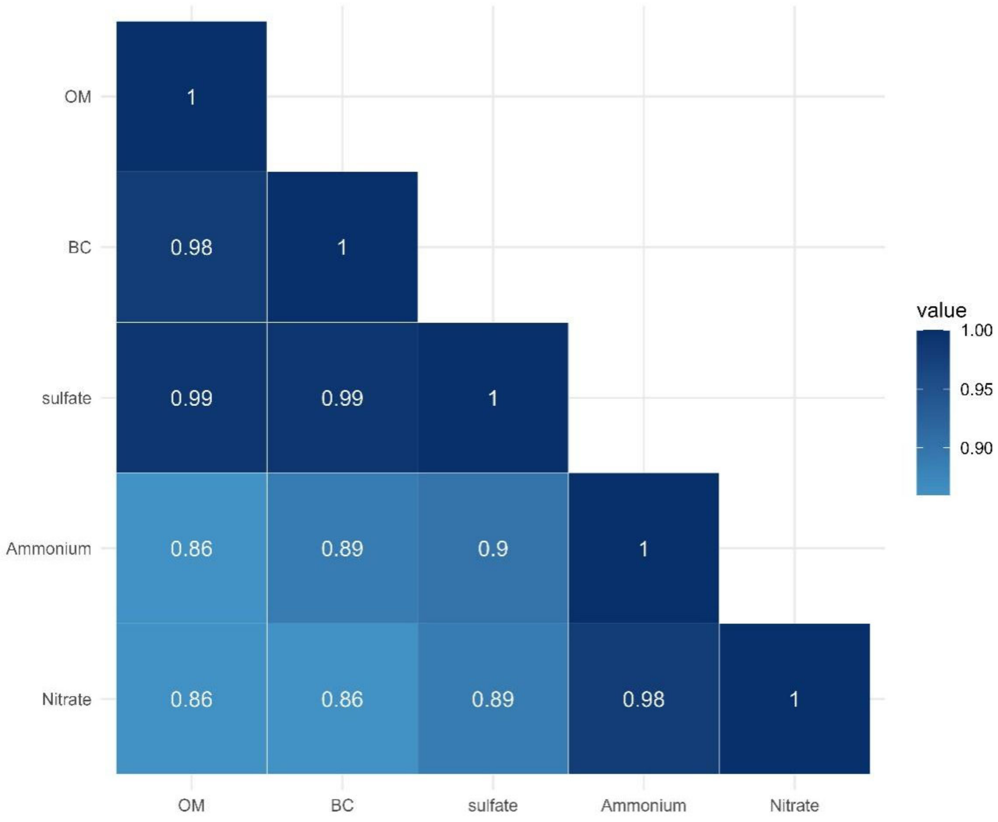

Figure S2 Pearson Correlation Coefficient Matrix Among the Five $PM_{2.5}$ Components (including organic matter (OM), black carbon (BC), sulfate (SO_4^{2-}), nitrate (NO_3^-), and ammonium (NH_4^+)).

7. The gWQS$_{int}$ model with a lower economic level as the reference

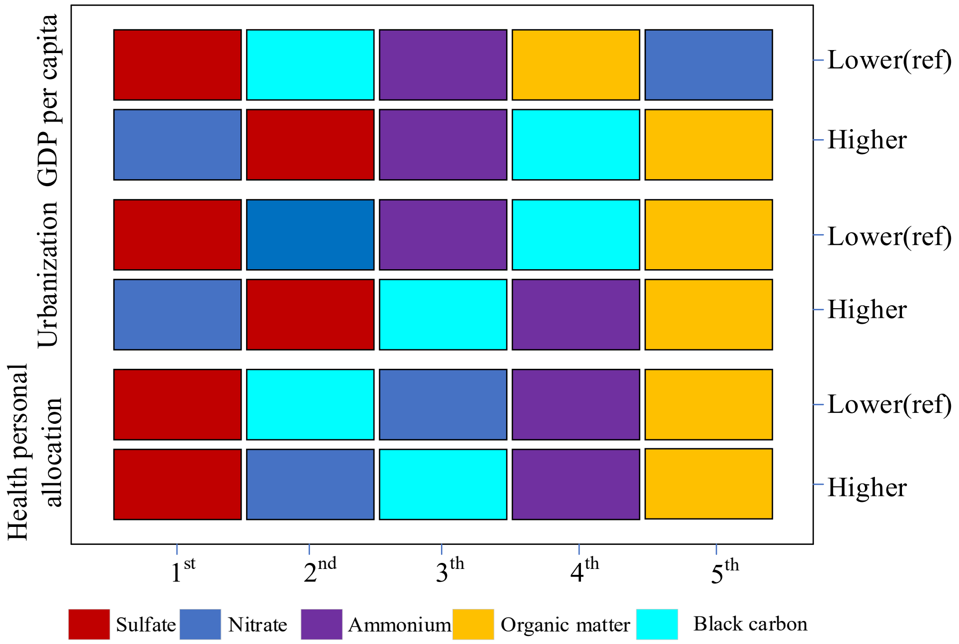

Figure S3 Ranking of Specific Stratified Weights (SSWs) for $PM_{2.5}$ Components (direction 2).
